# Wear Behavior of Al_2_O_3_/Ti_3_AlC_2_-MAX Composites at Elevated Temperatures

**DOI:** 10.3390/ma18163738

**Published:** 2025-08-10

**Authors:** Jaroslaw Wozniak, Wojciech Pawlak, Kamil Broniszewski, Tomasz Cygan, Anna Jędrzejczak, Andrzej Olszyna, Bartłomiej Przybyszewski

**Affiliations:** 1 Faculty of Material Science and Engineering, Warsaw University of Technology, ul. Wołoska, 141, 02-507 Warsaw, Poland; kamil.broniszewski@pw.edu.pl (K.B.); tomasz.cygan@pw.edu.pl (T.C.);; 2 Faculty of Mechanical Engineering, Lodz University of Technology, ul. Stefanowskiego 1/15, 90-924 Lodz, Poland

**Keywords:** tribology, sintering, composites, Ti_3_AlC_2_

## Abstract

This paper investigates the effect of Ti_3_AlC_2_-MAX phase addition as a solid lubricant to Al_2_O_3_. The composites were prepared using powder metallurgy and consolidated by Spark Plasma Sintering (SPS). The influence of Ti_3_AlC_2_ addition on phase composition and mechanical and tribological properties was evaluated. The use of MAX phase allows for the fabrication of sintered materials characterized by high density, a hardness close to 2000 HV5, and a fracture toughness of 5.5 MPa*m^0.5^ for the sample containing 10 wt.% Ti_3_AlC_2_. Moreover, a significant reduction in wear of the composites was observed for friction tests conducted at 700 °C compared to the unreinforced sample. The research demonstrates that Ti_3_AlC_2_ can serve as a solid lubricant and exhibits the best performance at high temperatures.

## 1. Introduction

Alumina is a widely used ceramic due to its impressive properties, including high hardness, good thermal and chemical stability, good wear resistance, and affordability. These advantages make it suitable for various applications such as electronics, power generation, and cutting tools [1,2,3,4]. A major limitation of using Al_2_O_3_ in cutting tools is its low fracture toughness. To improve this, composites based on aluminum oxide are produced. However, particular attention should be paid to the wear of these composites during friction at elevated temperature, which is extremely important in the case of these specific applications. Solid lubricants offer significant advantages over conventional lubricants, particularly in high-temperature applications (above 350 °C) and oxidizing environments. These advantages include better lubricity, good thermal stability, and good chemical stability. This highlights the need for continued exploration and development of solid lubricants for demanding operating conditions [5]. The following types of compounds are used as solid lubricants: polymer composites, soft metals, layered materials (graphite, hBN, MoS_2_), oxides (B_2_O_3_, TiO_2_, V_2_O_5_ and MoO_3_), and MAX phases (Ti_3_SiC_2_, Ti_2_AlC) [6,7,8,9]. One of the most promising due to its properties are MAX phases. They combine the properties of ceramics and metals [10]. MAX phases are a large and diverse group of crystalline materials with a layered structure. They are typically denoted by the formula M_n + 1_AX_n_, where M represents a light transition metal, A is a group 13 or 14 element, and X is carbon or nitrogen [11]. This layered structure contributes to MAX phases having properties of both ceramics and metals. Like ceramics, MAX phases exhibit high hardness, strength, and good thermal properties. Similar to metals, they show good electrical and thermal conductivity, high fracture toughness, and are relatively easy to machine [12]. These properties make MAX phases an ideal addition to ceramic matrix. Their high hardness will not significantly reduce the hardness of composites, while the flakelike structure will improve fracture toughness through mechanisms such as crack deflection and bridging. Moreover, due to their structure and properties, MAX phases can be used as an additive to ceramics to improve tribological properties at ambient and elevated temperatures.

The basic mechanism of MAX phase lubrication is very similar to that of graphite or MoS_2_. Due to the weak bonds between the M-X layers, they can slide over each other under relatively low shear stresses [13,14]. However, most layered materials lose their lubricating properties at elevated temperatures (above 350 °C) due to the degradation of their structure, especially in an oxidizing atmosphere. MAX phases have a much higher thermal stability. These materials retain their structure up to 600 °C in air [15] and over 1000 °C in a vacuum or inert gas [16]. MAX phases undergo oxidation during high-temperature friction. However, this process leads to the formation of a tribe-oxide film that significantly reduces material wear [17,18]. These properties have been confirmed in numerous scientific publications. Zhai et al. [19] investigated the tribological behavior of Ti_3_SiC_2_ compared to low-carbon steel. They employed a block-on-disc test method with a sliding speed of 20 m/s and applied loads corresponding to a stress range of 0.1 to 0.8 megapascals. The analysis revealed that the tribo-films formed on the surfaces were composed of TiO_2_, SiO_2_, and Fe_2_O_3_. El-Raghy et al. [20] investigated the grain size of Ti_3_SiC_2_ ceramic on tribological properties with the use of the pin-on-disk method. They showed that regardless of the grain size, the friction coefficient does not change significantly and is approximately 0.8. Sun et al. [21] investigated the tribological behaviour of a Ti_3_SiC_2_ ceramic containing 7 wt.% TiC against steel pins using a pin-on-disk tribometer. They showed no effect of normal load on COF and an increase in wear with increasing normal load. Moreover, they observed the formation of a tribofilm containing Fe. Hongxiang et al. [22] investigated the tribological properties of Ti_3_AlC_2_ ceramic. They employed a block-on-disc method and low-carbon steel as a counter sample. They obtained a very low COF of ∼0.1 and wear rate of ∼2.5 × 10^−6^ mm^3^/Nm, which they explained by the formation of a tribofilm containing Ti, O, Al and Fe.

Despite many publications on the tribological properties of MAX phase ceramics and composites based on them, there are no reports on the use of MAX phases as an additive to a ceramic matrix to improve tribological properties. The main objective of this article is to determine the influence of Ti_3_AlC_2_-MAX phase addition to Al_2_O_3_ on the improvement of tribological properties of composites both at room temperature and elevated temperature (700 °C) in air. The mechanisms responsible for the improvement of these properties will also be determined. Both the wear track of the sample and the counter-sample were subjected to detailed microscopic observations and chemical analysis. In addition, the basic mechanical properties of the composites were determined.

## 2. Materials and Methods

Table 1 provides a comprehensive overview of the fundamental characteristics of the powder substrates employed in the synthesis of the Ti_3_AlC_2_-MAX phase and the consolidation of Al_2_O_3_ matrix composites reinforced with MAX phase.

To prepare the MAX phases, titanium (GoodFellow, Cambridge, UK with a chemical purity of 99.6% and an average particle size below 20 µm), aluminum (Bend-Lutz Co. Skawina, Poland with a chemical purity of 99.7%), and carbon (Sigma-Aldrich, St. Louis, MO, USA with a chemical purity of 99.9% and the average particle size below 20 µm) powders were wet blended in isopropyl alcohol (no. 1759, Stanlab, Lublin, Poland) using a ball-type mill (Fritsch Pulverisette, Fritsch, Idar-Oberstein, Germany), then dried and sieved to #300 µm [23]. A molar ratio of Ti:Al:C = 3:1:1.9 was used. The reactive synthesis process was carried out using the SPS technique (FCT Systeme GMBH, Effelder Rauenstein, Germany) to synthesize the Ti_3_AlC_2_ MAX phases. The synthesis parameters were the following: a temperature of 1300 °C, a heating rate of 250 °C/min, and a vacuum atmosphere (*p* = 5 × 10^−2^ mbar). Following synthesis, the MAX phase was ground to a size below 45 µm using an automatic mortar grinder (Retsch KM100, Retsch GmbH, Haan, Germany), with a grinding bowl speed of 70 rpm and an applied force of 5–12.5 daN. In order to ensure the repeatability of the synthesis process, each batch of Ti_3_AlC_2_ was produced using powders from the same manufacturer with the same purity and particle size. Before each synthesis process, all powders were subjected to XRD analysis to verify the actual composition against the manufacturer’s declared specifications. Furthermore, great importance was attached to controlling the parameters of all manufacturing stages to minimize differences in the purity of the produced Ti_3_AlC_2_ powders. After drying and sieving, the powder mixture was synthesized using a Spark Plasma Sintering furnace (FCT HP D 10) under the following conditions: temperature: 1300 °C, heating rate: 250 °C/min, and vacuum atmosphere (*p* = 5 × 10^−2^ mbar); the powder was cooled immediately after reaching the maximum temperature. The synthesized Ti_3_AlC_2_-MAX phase was then ground with an automatic mortar grinder (Retsch KM100). The composites were fabricated using the powder metallurgy technique and consolidated via Spark Plasma Sintering (SPS). Initially, Al_2_O_3_-xTi_3_AlC_2_ powder mixtures, with varying weight percentages of Ti_3_AlC_2_ (x = 5, 10, 15, 20 wt.%), were wet-mixed using a horizontal mill for 24 h. Propan-2-ol and alumina grinding media (Nikkato, Osaka, Japan) were used during this step. Following drying, the powders were sieved (#300 µm). The composite mixtures were then sintered using Spark Plasma Sintering. The specific process parameters employed were the following: sintering temperature: 1400 °C, heating rate: 250 °C/min, dwell time: 4 min, applied pressure: 50 MPa, and vacuum atmosphere. As a reference, pure alumina samples were sintered under identical conditions. Fracture toughness (K_IC_) and hardness were evaluated using a Vickers Hardness Tester (FV-700e, Future Tech, Kanagawa, Japan) under a load of 49 N. Both measurements were performed on 3 samples, with at least 10 measurements taken on each. Friction tests were performed with the use of the high-temperature ‘pin-on-disc’ method (THT, CSM, Zurich, Switzerland). The friction radius was set at 4.5mm, linear velocity at 0.1 m/s, and load force at 1N. As a counter-sample, silicon nitride balls with a diameter of 6.35 mm were used. Two measurement temperatures were selected. For 20 °C, the test distances were 500 m (about 17,660 rotations) and 1000 m (about 35,320 rotations). For a high temperature of 700 °C, only a 500 m friction path was measured. The use of low linear velocity and low load force is associated with the potential use of these materials as cutting tools for materials that are difficult to process or require high precision, as well as for materials that are easily deformed. The selection of a temperature of 700 °C was associated with the higher thermal stability of the Ti_3_AlC_2_-MAX phase. The use of a lower temperature would not have caused the decomposition of the reinforcing phase during the friction test. The remaining friction test parameters such as linear velocity, load force, and friction distances were selected based on literature data for similar materials.

After the friction tests, the friction paths were measured with the use of optical profilometry (S-neox, Sensofar Metrology, Barcelona, Spain). In the case of the optical profiler studies, they were conducted in confocal mode. The objective magnification applied during the measurements was ×20. For each sample, eight different areas of the wear track were measured. Registered data, with dimensions of 850.08 × 709.32 µm^2^, were treated with the use of an academic license of OriginPro Version 2020 Software (OriginLab Corporation, Northampton, MA, USA). In the cases where the depth of the friction path was smaller than the surface roughness, the individual line profiles were analyzed to obtain proper worn volume estimation.

Integrated worn volumes in the friction paths were used to calculate the volumetric coefficients of wear from the following known formula:

Kw = V*F^−1^*S^−1^ (1)

where: Kw—coefficient of wear [mm^3^·N^−^^1^ · m^−^^1^], V—volume of wear [mm^3^], F—load force [N], S—test distance [m]. Based on the separate results, the average wear rate values were estimated for all composites.

Similarly, the wear coefficients for stationery ball counter-samples were appraised. The worn balls’ volume was determined by the diameter of the worn spherical bowl at a known radius of the balls used. Diameter measurements were made using SEM. Worn surfaces of the Al_2_O_3_ matrix composites and Si_3_N_4_ balls were examined using scanning electron microscopes (Axia ChemiSEM Thermo Scientific, Waltham, MA, USA and STEM Hitachi 5500, Tokyo, Japan). The degree of coverage of the wear track by the tribofilm was determined using image analysis. At least 10 images from different areas of the sample were used for the measurement. The images were analyzed using NIS Elements software 5. Phase analysis of the synthesized powders and consolidated composites was performed using a Rigaku, Tokyo, Japan MiniFlex II X-ray diffractometer with Cu Kα radiation (λ = 0.154059 nm). Additionally, the density of the Al_2_O_3_/MAX composites was measured using a helium pycnometer (Ultrapycnometer 1000 helium pycnometer, Quantachrome Instruments, Drive Boynton Beach, FL, USA).

## 3. Results

**Figure 1:**
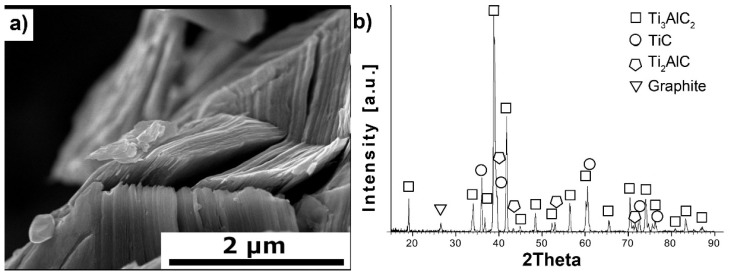
Characterization of synthesized Ti_3_AlC_2_-MAX phase powders, (**a**) morphology and (**b**) XRD analysis.

Figure 1a,b presents the morphology and phase composition of MAX powders used as a reinforcing phase for Al_2_O_3_-based composites. The morphology of the Ti_3_AlC_2_-MAX powders exhibits a layered structure with distinct flakes forming the powder particle. This indicates that the Ti_3_AlC_2_-MAX phase synthesis process was successful. Additionally, these observations are confirmed by the phase composition studies. The presence of the following four phases was observed: Ti_3_AlC_2_, TiC, Ti_2_AlC, and graphite. However, the most desired phase, Ti_3_AlC_2_, is the dominant phase, while the others are present in negligible amounts.

**Figure 2:**
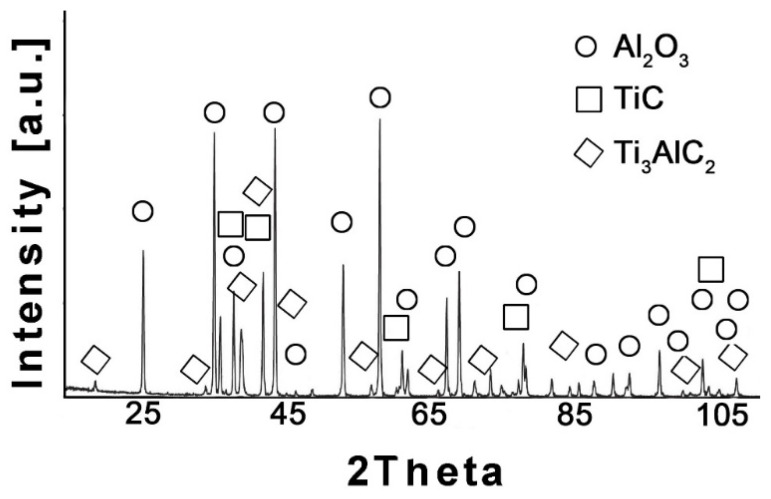
XRD analysis of Al_2_O_3_ + 20 wt.% Ti_3_AlC_2_ addition showing the presence of three phases: Al_2_O_3_, TiC, and Ti3AlC_2_.

The phase composition analysis was also performed for the Al_2_O_3_-based composites. The X-ray diffraction pattern for the Al_2_O_3_ composite with wt.% Ti_3_AlC_2_ is illustrated in Figure 2.

**Figure 3:**
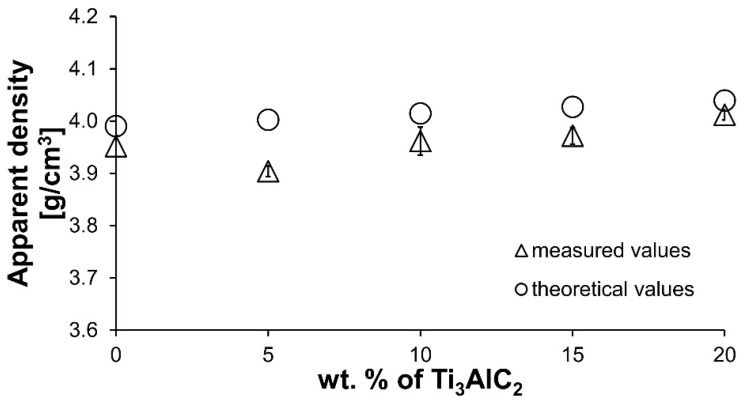
The experimental apparent density of Al_2_O_3_-Ti_3_AlC_2_ composites and the associated theoretical density.

Figure 3 presents the results of density measurements for composites with varying Ti_3_AlC_2_-MAX phase content. An initial decrease in density is observed for the composite with the lowest Ti_3_AlC_2_-MAX phase content, followed by an increase in density with increasing Ti_3_AlC_2_ content. The maximum apparent density was achieved for composites containing 20 wt.% MAX phase. The increase in sinter density is caused by the addition of a Ti_3_AlC_2_-MAX phase with a higher density compared to Al_2_O_3_. Relative density was not determined due to the presence of TiC in the sintered structure and the associated difficulties in determining the correct theoretical density of the sintered samples. Only the theoretical density for composites containing pure Ti_3_AlC_2_ is presented. The measured values exhibit the same increasing trend as the theoretical values.

**Figure 4:**
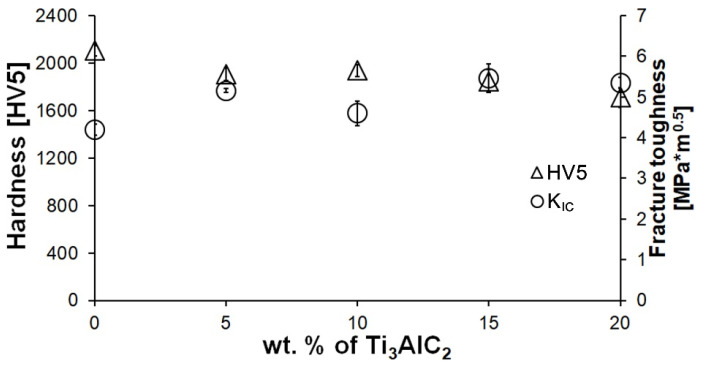
Hardness (HV5) and fracture toughness (K_IC_) of Al_2_O_3_-Ti_3_AlC_2_ composites with varying Ti_3_AlC_2_ content.

A decrease in hardness is observed for the composites in comparison to the reference sample (Figure 4). Moreover, the increase in the Ti_3_AlC_2_-MAX phase fraction does not significantly affect the hardness value. Only a slight decrease in hardness was observed with increasing Ti_3_AlC_2_ content. Much larger differences were observed in the case of fracture toughness (Figure 4). Even the smallest addition of MAX phase significantly increases K_IC_. However, no significant improvement in K_IC_ is observed with increasing MAX phase fraction.

**Figure 5:**
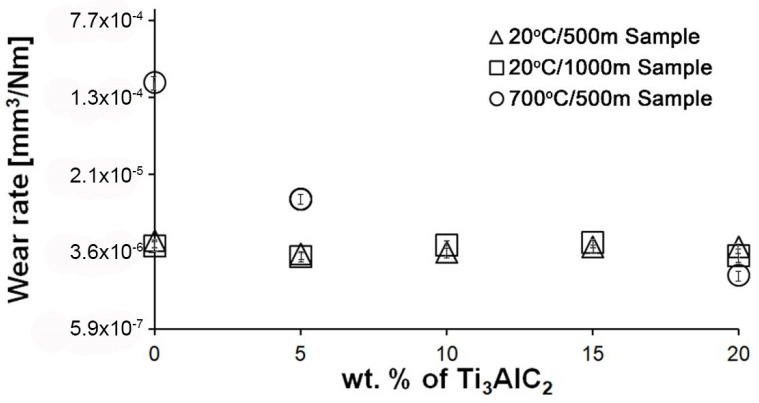
Influence of Ti_3_AlC_2_-MAX phase content on wear rate of the Al_2_O_3_ matrix composites at different wear test conditions.

Figure 5 shows the wear of composites as a function of MAX phase fraction for two temperatures and friction paths. In the case of samples tested at room temperature, no significant change in wear was observed with the change in the volume fraction of the reinforcing phase. Doubling the friction distance also did not significantly affect the wear value. Differences are visible in the case of the test conducted at 700 °C. There is a significant decrease in wear of Al_2_O_3_ − 5% Ti_3_AlC_2_-MAX phase compared to the reference sample. In the samples with 10 and 15% of Ti_3_AlC_2_-MAX phase after the high-temperature friction tests, the build-up formation was observed. The formation of the build-up prevented wear measurement, which explains the lack of points for the 10 and 15% of Ti_3_AlC_2_-MAX phase samples in Figure 5. The maximum volume fraction of the reinforcing phase allowed for an even lower wear value compared to the sample with 5% Ti_3_AlC_2_-MAX phase content.

**Figure 6:**
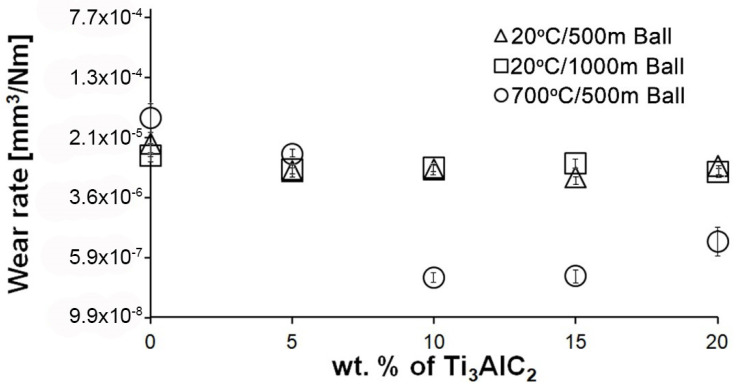
Wear of Si_3_N_4_ counterparts working with composites with different Ti_3_AlC_2_ content at different wear test conditions.

An almost identical wear profile was observed for the Si_3_N_4_ counterparts (Figure 6). For samples tested at room temperature, the wear value is almost identical regardless of the friction path and the MAX phase fraction in the composite. In the case of the test conducted at 700 °C, similar to the composites, the addition of Ti_3_AlC_2_-MAX phase reduces the wear of the counterpart. Lower wear values were observed for samples with 10% and 15% MAX, for which the formation of a build-up on the surface of the composites was observed. For the counterpart working with the composite containing 20% MAX, a slight increase in the wear value was observed.

**Figure 7:**
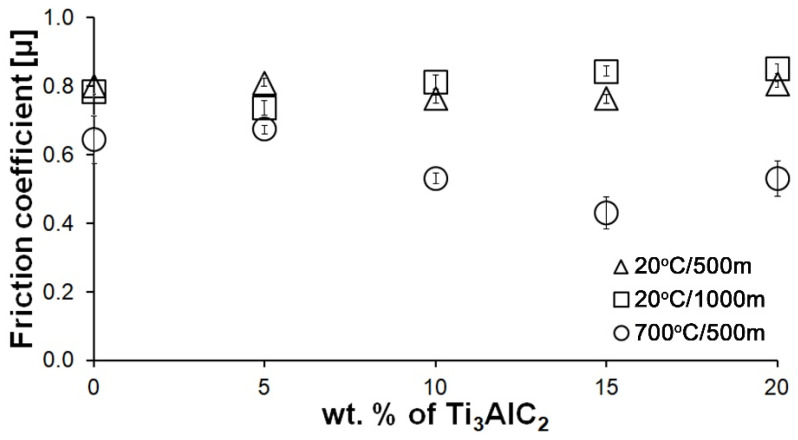
Influence of Ti_3_AlC_2_ content on the friction coefficient of Al_2_O_3_-based composites.

Figure 7 shows the average friction coefficient values for all composites and tests conditions. Again, no significant changes were observed for the tests conducted at room temperature. Similar average friction coefficient values are observed for all samples. However, in the case of the test conducted at elevated temperature, a decrease and then an increase in the average friction coefficient value is observed for composites containing 10, 15, and 20% Ti_3_AlC_2_-MAX phase, respectively. This change is identical to that obtained for wear rate.

**Figure 8:**
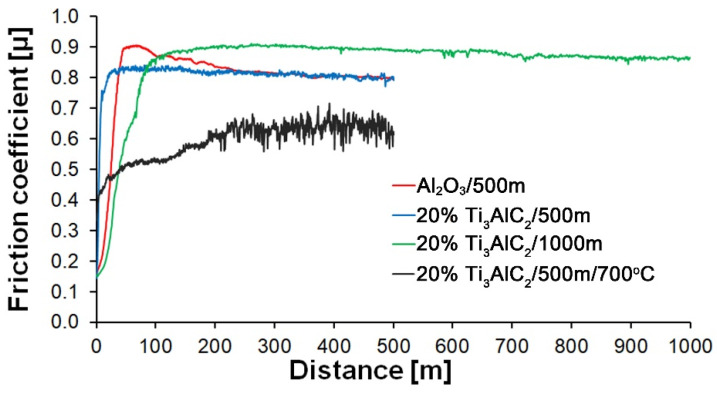
Effect of sliding distance on friction coefficient of Al_2_O_3_ and Al_2_O_3_ + 20 wt.% Ti_3_AlC_2_ at different wear test conditions.

Furthermore, the analysis of the friction coefficient changes as a function of the sliding distance reveals significant differences between the tested materials (Figure 8). The reference sample shows an increase in the friction coefficient to about 0.9, followed by a slow decrease and stabilization of its value after about 300 m. In contrast, no initial running stage is observed for the composites. The friction coefficient maintains a stable value throughout the entire test. For the samples tested at 700 °C, the friction coefficient is lower than the others and fluctuates significantly after reaching its maximum value.

**Figure 9:**
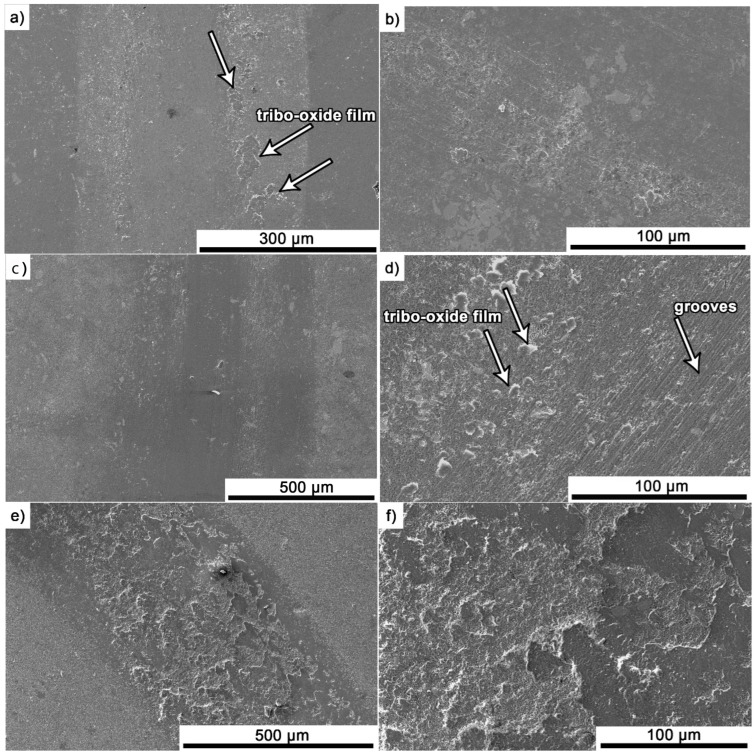
Scanning electron microscopy (SEM) images of the worn surface of Al_2_O_3_ + 5 wt.% Ti_3_AlC_2_: (**a**,**b**) 20 °C/500m, (**c**,**d**) 20 °C/1000 m, (**e**,**f**) 700 °C/500 m.

After the friction test, the surface of the samples was analysed using a scanning electron microscope. Exemplary images for composites containing 5% Ti_3_AlC_2_-MAX phase are presented in Figure 9a–f. For composites tested at room temperature (Figure 9a–d), a similar wear pattern was observed. The wear surface is relatively smooth with visible shallow grooves. Additionally, the presence of tribo-oxide films was observed. However, these areas are rather isolated and do not cover the entire wear track. The measured tribofilm coverage values for the wear surface are 5.5% and 7% for the samples Al_2_O_3_ + 5 wt.% Ti_3_AlC_2_ 20 °C/500 m and Al_2_O_3_ + 5 wt.% Ti_3_AlC_2_ 20 °C/1000 m, respectively. A completely different surface is observed for composites tested at elevated temperature (Figure 9e,f). For these samples, almost the entire wear surface (96%) is covered with a tribo-oxide film that cracks and spalling during friction.

**Figure 10:**
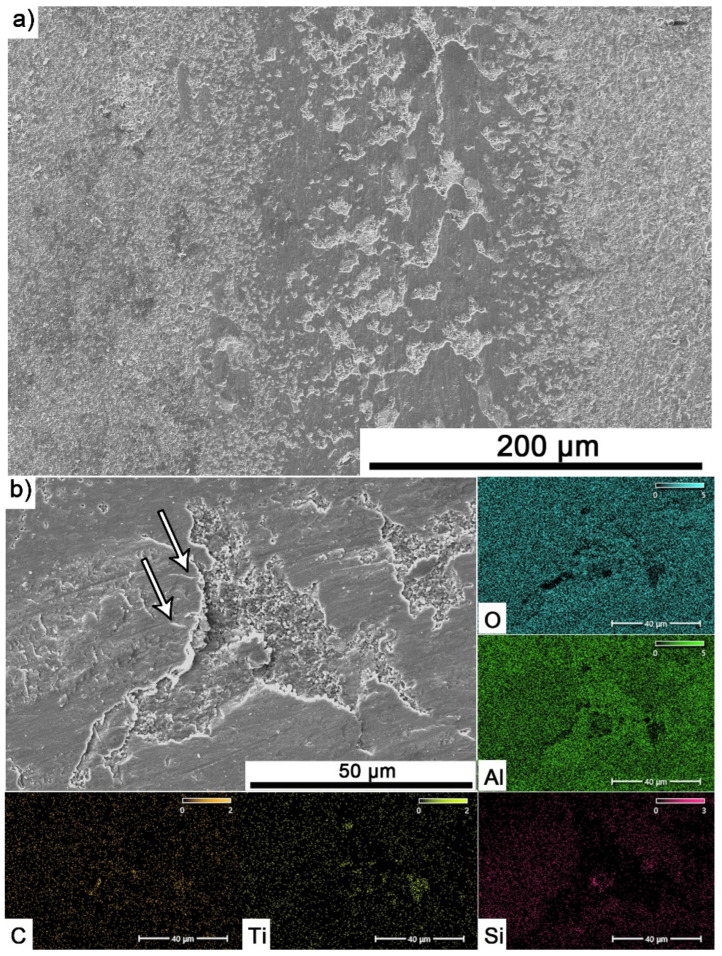
SEM images with visible build-up for Al_2_O_3_ + 10 wt.% Ti_3_AlC_2_ specimen tested at 700 °C and 500 m sliding distance: (**a**) microstructure and (**b**) chemical composition.

Elemental analysis of the surfaces with visible build-up for the composite containing 10% MAX tested at 700 °C (Figure 10a,b) showed the presence of mainly Al and O, which confirms the formation of a tribo-oxide film. The presence of Ti originating from the MAX phase or TiC and Si from the counter sample was also observed.

**Figure 11:**
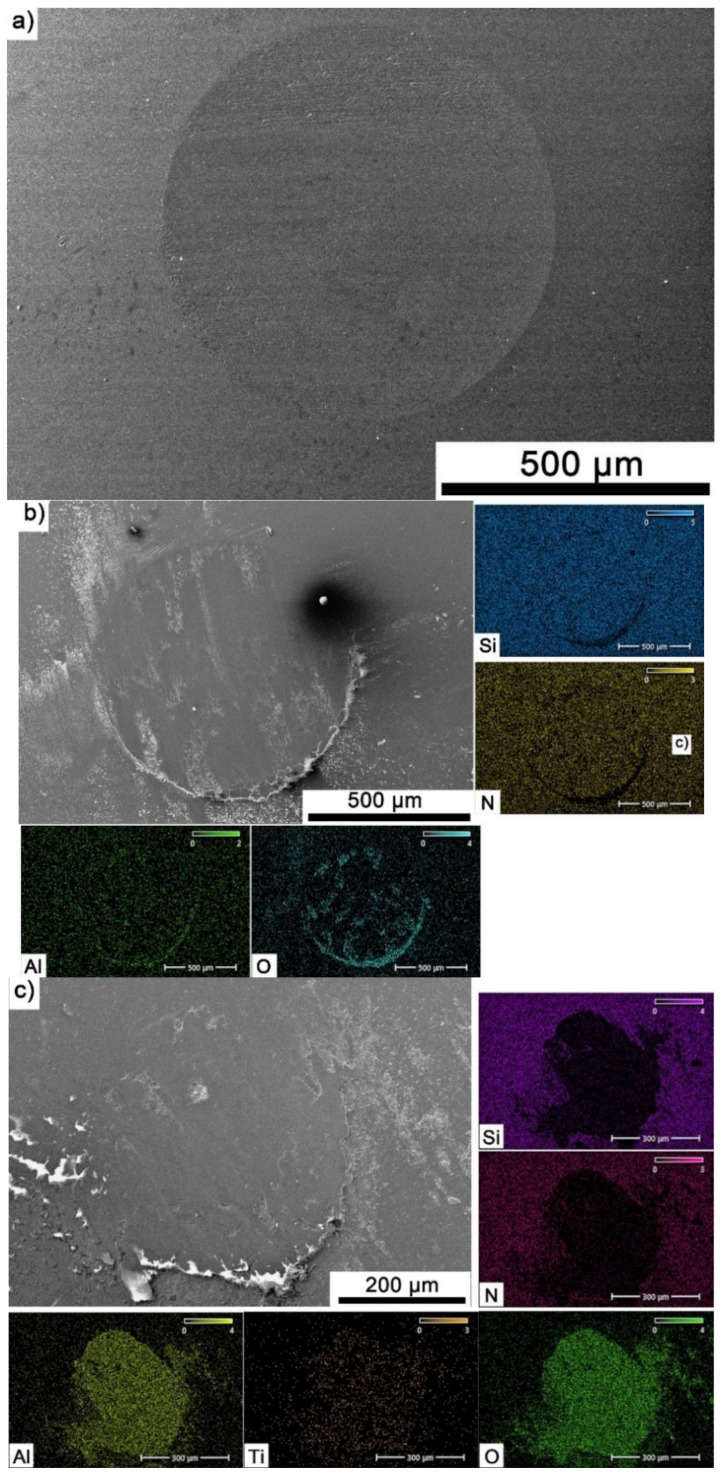
Microstructure and chemical composition of the worn surface of Si_3_N_4_ balls after tribological testing, (**a**) Al_2_O_3_—20 °C/500 m, (**b**) Al_2_O_3_ + 20 wt.% Ti_3_AlC_2_—20 °C/500 m, (**c**) Al_2_O_3_ + 20 wt.% Ti_3_AlC_2_—700 °C/500 m.

The Si_3_N_4_ balls were also subjected to observation and EDS analysis. Figure 11a–c showed exemplary wear tracks for counter-samples that were a friction pair with reference sample and composites containing 20% Ti_3_AlC_2_-MAX phase. For Al_2_O_3_ (Figure 11a), the counter-samples exhibit uniform smooth wear with few grooves. Increasing the reinforcement content (Figure 11b) resulted in the appearance of a tribo-oxide film on the counter-sample surface, containing mainly Al_2_O_3_. In the case of the counter-sample tested at 700 °C (Figure 11c), a tribo-oxide film is also present, but its thickness is much larger and its elemental composition includes titanium, which is a product of the decomposition of Ti_3_AlC_2_-MAX phase.

## 4. Discussion

The comparison of the phase composition of the MAX phase powder, and the consolidated composite shows that the use of the SPS sintering method prevented the decomposition of the MAX phase during the consolidation process (Figure 1b and Figure 2). The XRD analysis of the composites revealed the presence of mainly Ti_3_AlC_2_ and an addition of TiC. The presence of titanium carbide was also reported by other authors [24]. It is related to the TiC residues from the MAX phase synthesis stage. Additionally, due to the lack of the Ti_2_AlC phase in the composites, TiC may be a product of the decomposition of this phase during the sintering process. Pang et al. [25] showed that Ti_2_AlC can decompose above 1400 °C in vacuum to Al and TiC_x_. Moreover, partial decomposition of Ti_3_AlC_2_ phases may also occur during the sintering process. All these factors cause TiC to be present in the consolidated composite. However, due to the high heating and cooling rates and the short sintering time used in the SPS method, the degradation of the strengthening phase is significantly limited [26]. The positive effect of the applied consolidation method is also visible in the density values obtained for the composites. As mentioned earlier, the relative density was not determined due to the difficulties in determining the correct theoretical density. However, based on the presented theoretical density values, calculated assuming the use of pure Ti_3_AlC_2_, it can be concluded that the addition of Ti_3_AlC_2_ does not significantly reduce the density of the obtained sinters (Figure 3). The measured values are slightly lower compared to the theoretical values, indicating low porosity and a negligible amount of the TiC phase, which has a higher density than Ti_3_AlC_2_. This is also confirmed by studies of the mechanical properties of the composites. The addition of Ti_3_AlC_2_, whose hardness is much lower (HV 380 [27]) than that of the Al_2_O_3_ matrix, did not cause a significant decrease in the hardness of the composites compared to the reference sample. The hardness value decreased by only 9%. Moreover, only a slight decrease in hardness was observed with increasing reinforcement content. Comparing the sample with the highest hardness (10% Ti_3_AlC_2_) with the sample with the lowest hardness (20% Ti_3_AlC_2_), the decrease was only 12%. As already mentioned, this decrease is related to the addition of a phase with lower hardness and the generation of pores at the matrix-reinforcing phase interface. In the case of fracture toughness, an opposite trend is observed (Figure 4). The addition of a reinforcing phase with a higher K_IC_ value (approximately 6.4 MPa*m0.5 [28]) resulted in a 25% improvement in this parameter compared to the reference sample. This increase results from a change in the fracture mechanism of the composites. According to literature data, crack propagation in the Ti_3_AlC_2_-MAX phase is more tortuous due to its layered structure. This leads to an extended propagation path and a reduced crack tip stress [28].

As the presented research results show, the addition of Ti_3_AlC_2_ to the alumina matrix has a positive effect on the tribological properties of composites. The positive influence of this reinforcing phase is more significant for materials working at high temperatures. Similar trends of wear values are observed for the samples tested at room temperature (Figure 5). The sample containing 5% Ti_3_AlC_2_-MAX phase exhibits a slight decrease in wear, which increases with the increasing volume fraction of the reinforcing phase. Lower wear values may be caused both by changes in the mechanical properties of the sintered materials and by the formation of tribo-oxide films (Figure 9a–d). The presence of tribo-oxide films, on the one hand, improves tribological properties by reducing wear, but on the other hand, can lead to the detachment of their fragments and abrasive wear of the tribo-pair [29]. In the case of samples tested at room temperature, the tribo-oxide films in the form of a single areas were observed; worn surface analysis showed that the number of these areas is greater for samples after a longer friction period. This should translate into lower wear; however, due to the fact that the tribo-oxide film areas are single, they are more easily detached from the surface, which causes higher abrasive wear. This is confirmed by studies of the wear surface, which show a larger number of grooves for samples after 1000 m compared to 500 m (Figure 9c,d). This is also reflected in the average coefficient of friction (Figure 7). An increase in this parameter is observed with increasing volume fraction of the reinforcing phase, which is associated with the formation of tribo-oxide films and a greater contribution of abrasive wear. Analyzing the changes in the coefficient of friction as a function of the path, significant differences are visible between the composites and the reference sample (Figure 8). In the case of pure Al_2_O_3_, an increase in the friction coefficient is observed during the initial meters of the tests. This stage is attributed to the removal of roughness from the contacting surfaces. Subsequently, the friction coefficient decreases and reaches a steady-state level [14]. In the case of composites, no running-in range was observed. The coefficient of friction reaches a constant maximum value from the beginning of the friction process. This is related to the presence of Ti_3_AlC_2_ and the formation of tribo-oxide films on both the composite surface and the counter-sample surface (Figure 11a,b).

Significantly larger differences between the composites and the reference sample are visible for tests conducted at 700 °C. A very high increase in wear was observed for pure Al_2_O_3_ sintered at room temperature. The addition of MAX phase reduces wear to the level of measurements at room temperature. This is related to the formation of a tribo-oxide film that covers almost the entire wear surface (Figure 9c,f). The effect of the film formation is a significant reduction in the wear of the composites. Analysis of the chemical composition of the film (Figure 10a,b) confirms that the film consists mainly of Al_2_O_3_. Trace amounts of Si are associated with the embedding of wear products from the Si_3_N_4_ counter-sample. In addition, the presence of carbon and titanium was identified in the same places. This indicates the degradation of Ti_3_AlC_2_-MAX phase during the friction process and the formation of TiC [29]. Although Ti_3_AlC_2_ is stable at 700 °C, the low thermal conductivity of Al_2_O_3_ can lead to an increase in temperature on the surface of the samples, which in turn will lead to the degradation of the MAX phase [23]. The wear of the counter-samples also shows a significant decrease with increasing MAX phase content (Figure 6) compared to the reference sample. This is related to the formation of a thick tribo-oxide film on its surface (Figure 11c). This film is significantly thicker compared to samples tested at room temperature. Similarly to the film on the composites, it also consists mainly of Al_2_O_3_ with trace amounts of titanium from the degradation of MAX phase. The coefficient of friction also changed. Average coefficient of friction values are significantly lower than for samples tested at room temperature (Figure 7). In addition, the change in this coefficient as a function of the friction path shows a different course (Figure 8). A slower increase in its value is observed, followed by an area of significant fluctuations in its value. This is related to the processes taking place on the surface of the sample. Figure 10b shows fine cracks in the tribo-oxide film, indicating that the film cracks and crumbles during friction. This creates discontinuities in the tribo-oxide film, which are the cause of large fluctuations in the coefficient of friction. It should be noted, however, that the applied load force 1N is relatively low compared to those typically used for ceramic materials. It is not possible to definitively determine whether the same mechanisms will occur at higher loads. Further research on the friction of Al_2_O_3_-based composites reinforced with Ti_3_AlC_2_ is necessary.

A detailed analysis of the worn surfaces reveals that the dominant wear mechanisms differ significantly between room temperature and elevated temperature conditions. At 20 °C, the primary mechanism is abrasive wear, evidenced by the presence of shallow grooves and isolated tribo-oxide films that are prone to detachment. In contrast, at 700 °C, the main mechanism shifts to oxidative wear and the formation of a strong, continuous tribofilm, which covers nearly the entire surface (96%). This protective layer is responsible for the significant reduction in wear and the lower, yet fluctuating, friction coefficient observed at high temperatures. The observed fluctuations are a result of the cracking and spalling of this tribo-oxide film during the friction process.

Table 2 summarizes the obtained results for the friction coefficient and wear rate with literature data. The comparison was made for tests performed at room temperature. It is clearly visible that composites containing Ti_3_AlC_2_ exhibit significantly lower wear (by about 5 orders of magnitude) compared to multi-layered graphene (MLG), multi-layered graphene coated with Ni-P (MLG-Ni-P), or graphene oxide (GO). Smaller differences are visible for the hBN-reinforced composite. However, in this case as well, the wear value for composites reinforced with Ti_3_AlC_2_ is one order of magnitude lower. The opposite character was observed for the friction coefficient. For the composite containing Ti_3_AlC_2_, the friction coefficient is about two times higher compared to the mentioned materials. However, it should be noted that, as shown by the obtained test results, the friction coefficient decreases significantly with increasing temperature. For all the mentioned materials, the thermal stability is similar. The degradation process for friction tests conducted in air begins at a temperature of 800–900 °C. However, it should be noted that the friction test conditions for the compared materials are different, therefore comparative tests for these materials conducted under the same friction conditions are necessary.

## 5. Conclusions

This study successfully demonstrates that adding the Ti_3_AlC_2_-MAX phase to an Al_2_O_3_ matrix significantly improves the composite’s mechanical and tribological properties. Key findings indicate that although the reinforcement has little effect on hardness, it significantly improves fracture toughness. The most pronounced tribological benefits are observed at elevated temperatures (700 °C), where degradation of the MAX phase leads to the formation of a strong, continuous tribo-oxide layer that dramatically reduces wear and stabilizes the coefficient of friction. This tribo-oxide layer also occurs on the Si_3_N_4_ counter-sample, further reducing wear across the entire tribological pair.

This study highlights the potential of Ti_3_AlC_2_-MAX phases as effective solid lubricants in high-temperature applications. However, the study also reveals a number of limitations. The observed degradation of the MAX phase at 700 °C suggests that the stability of the composites at temperatures above this threshold requires further investigation. Furthermore, the results are limited to specific loading conditions, and future work should encompass a broader range of parameters to fully understand the material’s properties under various operating scenarios. These results provide a foundation for the development of high-performance Al_2_O_3_-based composites for applications in demanding high-temperature environments, although a more comprehensive understanding of their long-term stability and broader applicability is still needed.

## Figures and Tables

**Table 1:** Parameters of powder substrates used for MAX phase synthesis and composite consolidation.

Powder	Purity	APS *	Manufacturer
Alumina	99.99%	0.125 µm	Taimei Chemicals Co., Ltd., Tokyo, Japan
Titanium	99.6%	<20 µm	GoodFellow, Cambridge, UK
Aluminum	99.7%	6.74 µm	Bend-Lutz Co., Skawina, Poland
Synthetic graphite	99.9%	<20 µm	Sigma-Aldrich, St. Louis, MO, USA

* average particle size.

**Table 2:** Comparison of the friction coefficient and wear rate of the obtained materials with literature data (for a temperature of 25 °C).

Material	Friction Coefficient [μ]	Wear Rate [mm^3^/Nm]	Ref.
Al_2_O_3_ + 40 wt % hBN	0.33	2.22 × 10^−5^	[30]
Al_2_O_3_ + 2 vol % MLG	0.46	6 × 10^−2^	[14]
Al_2_O_3_ + 1 vol % MLG/Ni-P	0.4	2.5 × 10^−1^	[14]
Al_2_O_3_ + 0.2 wt % GO	-	4.8 × 10^−1^	[31]
Al_2_O_3_ + 20 wt % Ti_3_AlC_2_	0.85	3.26 × 10^−6^	

## Data Availability

The raw data and processed data required to reproduce these findings cannot be shared at this time because it is part of ongoing studies but may be available upon request.

## References

[1] Koli D.K., Agnihotri G., Purohit R. (2014). A Review on Properties, Behaviour and Processing Methods for Al-Nano Al_2_O_3_ Composites. Procedia Mater. Sci..

[2] Munro M. (1997). Evaluated Material Properties for a Sintered Alpha-Alumina. J. Am. Ceram. Soc..

[3] Sternitzke M. (1997). Structural ceramic nanocomposites. J. Eur. Ceram. Soc..

[4] Niihara K. (1991). New Design Concept of Structural Ceramics. J. Ceram. Soc. Jpn..

[5] Marques A., Suarez M.P., Sales W.F., Machado R. (2018). Turning of Inconel 718 with whisker-reinforced ceramic tools applying vegetable-based cutting fluid mixed with solid lubricants by MQL. J. Mater. Process. Technol..

[6] Torres H., Ripoll M.R., Prakash B. (2018). Tribological behavior of self-lubricating materials at high temperatures. Int. Mater. Rev..

[7] Wang Y., Liu X.B., Liu Y.F., Luo Y.S., Meng Y. (2020). Microstructure and tribological performance of Ni60-based composite coatings on Ti6Al4V alloy with different Ti_3_SiC_2_ ceramic additions by laser cladding. Ceram. Int..

[8] Aouadi S.M., Luster B., Kohli P., Muratore C., Voevodin A.A. (2009). Progress in the development of adaptive nitride-based coatings for high temperature tribological application. Surf. Coat. Technol..

[9] Petrus M., Wozniak J., Cygan T., Kostecki M., Cygan S., Jaworska L., Teklińska D., Olszyna A. (2019). Comprehensive study on graphene-based reinforcements in Al_2_O_3_–ZrO_2_ and Al_2_O_3_–Ti(C,N) systems and their effect on mechanical and tribological properties. Ceram. Int..

[10] Barsoum M.W., Radovic M. (2011). Elastic and mechanical properties of the MAX phases. Annu. Rev. Mater. Res..

[11] Wozniak J., Jastrzębska A., Olszyna A. (2022). Challenges and opportunities in tailoring MAX phases as a starting materials for MXenes development. Mater. Technol..

[12] Lange C., Barsoum M.W., Schaaf P. (2007). Towards the synthesis of MAX-phase functional coatings by pulsed laser deposition. Appl. Surf. Sci..

[13] Magnus C., Cooper D., Sharp J., Rainforth W.M. (2019). Microstructural evolution and wear mechanism of Ti_3_AlC_2_-Ti_2_AlC dual MAX phase composite consolidated by spark plasma sintering (SPS). Wear.

[14] Wozniak J., Cygan T., Petrus M., Cygan S., Kostecki M., Jaworska L., Olszyna A. (2018). Tribological performance of alumina matrix composites reinforced with nickel-coated graphene. Ceram. Int..

[15] Prikhna T., Ostash O., Sverdun V., Karpets M., Zimych T., Ivasyshin A., Cabioc’h T., Chartier P., Dub S., Javorska L. (2018). Presence of oxygen in Ti-Al-C MAX phases-based materials and their stability in oxidizing environment at elevated temperatures. Acta Phys. Pol. A.

[16] Low I.M., Pang W.K., Kennedy S., Smith R.I. (2011). High-temperature thermal stability of Ti_2_AlN and Ti_4_AlN_3_. A comparative diffraction study. J. Eur. Ceram. Soc..

[17] Meng J., Lu J., Wang J., Yang S. (2006). Tribological behavior of TiCN-based cermets at elevated temperatures. Mater. Sci. Eng. A.

[18] Maslyuk V.A. (2003). Tribological properties of hard alloys based on chromium carbide. Powder Metall. Met. Ceram..

[19] Zhai H.Z., Huang Z.Y., Zhou Y., Zhang Z.L., Wang Y., Al M. (2004). Oxidation layer in sliding friction surface of high-purity Ti_3_SiC_2_. J. Mater. Sci..

[20] El-Raghy T., Blau P., Barsoum M.W. (2000). Effect of grain size on friction and wear behavior of Ti_3_SiC_2_. Wear.

[21] Sun Z.M., Zhou Y.C., Li S. (2002). Tribological behavior of Ti3SiC2-based material. J. Mater. Sci. Technol..

[22] Hongxiang Z., Zhenying H., Zingxing A., Yang Z., Zhilli Z., Shibo L. (2005). Tribophysical properties of polycrystalline bulk Ti_3_AlC_2_. J. Am. Ceram. Soc..

[23] Petrus M., Wozniak J., Cygan T., Pawlak W., Olszyna A. (2022). Novel alumina matrix composites reinforced with MAX phases—Microstructure and mechanical properties. Materials.

[24] Zheng L., Li F., Zhou Y. (2012). Preparation, microstructure, and mechanical properties of TiB_2_ using Ti_3_AlC_2_ as a sintering aid. J. Am. Ceram. Soc..

[25] Pang W.K., Low I.M., O’Connor B.H., Peterson V.K., Studer A.J., Palmquist J.P. (2011). In situ diffraction study of thermal decomposition in Maxthal Ti_2_AlC. J. Alloys Compd..

[26] Guillard F., Allemand A., Lulewicz J.D., Galy J. (2007). Densification of SiC by SPS-effects of time, temperature and pressure. J. Eur. Ceram. Soc..

[27] Wang W., Li C., Zhai H., Wang C. (2015). Preparation of High-Strength Ti_3_AlC_2_ by Spark Plasma Sintering. J. Appl. Ceram. Technol..

[28] Yao L., Zhu C.C., Jiang J.X., Zhou B.B. (2022). Mechanical properties of Ti_3_AlC_2_ ceramics before and after heat treatment. Rare Met..

[29] Dang W., Ren S., Zhou J., Yu Y., Wang L. (2016). The tribological properties of Ti_3_SiC_2_/Cu/Al/SiC composite at elevated temperatures. Tribol. Int..

[30] Sun Q., Song J., Li J., Lin P., Dong Y., Su Y., Fan H., Hu L., Zhang Y. (2025). Tribological behavior of h-BN/Al_2_O_3_ self-lubricating composites in extreme environments–Part I: Lubricating behavior and ultra-low friction coefficient mechanism in air environments from room temperature to 1200 °C. Tribol. Int..

[31] Cygan T., Petrus M., Wozniak J., Cygan S., Teklińska D., Kostecki M., Jaworska L., Olszyna A. (2020). Mechanical properties and tribological performance of alumina matrix composites reinforced with graphene-family materials. Ceram. Int..

